# Combined effects of elevated temperature and CO_2_ enhance threat from low temperature hazard to winter wheat growth in North China

**DOI:** 10.1038/s41598-018-22559-4

**Published:** 2018-03-12

**Authors:** Kaiyan Tan, Guangsheng Zhou, Xiaomin Lv, Jianping Guo, Sanxue Ren

**Affiliations:** 1 State Key Laboratory of Severe Weather, Chinese Academy of Meteorological Sciences, Beijing, 100081 China; 2 Collaborative Innovation Center on Forecast Meteorological Disaster Warning and Assessment, Nanjing University of Information Science & Technology, Nanjing, 210044 China

## Abstract

We examined the growth and yield of winter wheat (*Triticum aestivum*) in response to the predicted elevated CO_2_ concentration and temperature to determine the mechanism of the combined impacts in North China Plain. An elevated treatment (CO_2_: 600 μmol mol^−1^, temperature: +2.5~3.0 °C, ECTI) and a control treatment (ambient CO_2_ and temperature, CK) were conducted in open-top chambers from October 2013 to June 2016. Post-winter growth stages of winter wheat largely advanced and shifted to a cooler period of nature season under combined impact of elevated CO_2_ and temperature during the entire growing season. The mean temperature and accumulated photosynthetic active radiations (PAR) over the post-winter growing period in ECTI decreased by 0.8–1.5 °C and 10–13%, respectively compared with that in CK, negatively impacted winter wheat growth. As a result, winter wheat in ECTI suffered from low temperature hazards during critical period of floret development and anthesis and grain number per ear was reduced by 10–31% in the three years. Although 1000-kernel weight in ECTI increased by 8–9% mainly due to elevated CO_2_, increasing CO_2_ concentration from 400 to 600 μmol mol^−1^ throughout the growth stage was not able to offset the adverse effect of warming on winter wheat growth and yield.

## Introduction

The impacts of climate change on crop production are becoming international concerns. They are particularly significant for the sustainable agricultural development and food supply. Wheat (*Triticum aestivum* L.) is one of the world’s most widely grown food crops, comprising more than one fourth of the global grain yield. Over the past decades, global climate change has exerted impact on wheat production^1–5^. Models that link yields of wheat to weather indicated wheat production declined by 5.5% from 1980 to 2008, relative to a counterfactual without climate trends in a majority of the wheat-growing countries^3^ and wheat production is estimated to fall by 6% for each °C of further temperature increase^1^. It is projected that global average temperature will rise by 3.7–4.8 °C by the end of the 21st century^6^. Increases in atmospheric CO_2_ concentration and associated further warming within the 21st century are likely to significantly affect future wheat production around world and crop hazard environmental factors.

Temperature and CO_2_ concentration are two of the key factors affecting crop development, growth and yield. Individual or combined effects of temperature rise and CO_2_ concentration change on wheat growth and yield during the recent decades have been examined^7–9^. But experiments on the combined effects were conducted mostly in controlled environment facilities or in temperature gradient tunnel with fixed day and night temperatures or a temperature increase occurring only during day time^10–14^, which is inconsistent with the observed temperature regime in nature and the temperature increasing trait from global warming. These studies improved understanding with regards to the interaction of higher CO_2_ concentration and temperature rise, but do not necessarily predict the accurate responses of wheat to the future environmental changing. Especially, the present study has paid less attention to the changes of crop hazard environmental factors under future climate change. It would be meaningful to design an experiment to investigate the responses of winter wheat to elevated CO_2_ concentration and matched temperature increment under a climate change scenario with ambient background conditions.

It is well known that elevated CO_2_ concentration stimulates crop biomass accumulation and increases crops yield for C_3_ plants such as wheat^7–9^, whereas the stimulating effect of CO_2_ enrichment depends on temperatures of growth environment^15,16^. Previous research proved that stimulation of photosynthesis with CO_2_ enrichment increased as temperature increased^17^. Therefore the stimulating effect of elevated CO_2_ on crop growth and yield might vary with climate warming in the future. Though temperature increase might reduce frost/chilling in wheat, it might also shorten growing period and cause heat stress during late growth stages leading to spikelet sterility and shortening grain filling duration^18,19^. The negative effects of warming were usually observed in experiments with spring wheat or with short period of warming^20–23^. Mitchell *et al*.^24^ found that warming throughout a growing season dramatically reduced the grain numbers per ear (37%) and yield (18%) of winter wheat. However, similar warming experiments on winter wheat showed different results^25–28^. These reports demonstrated the warming effects are more complicated and depend on cultivars (spring or winter), the magnitude of warming and geographical location.

The North China Plain is one of the most important regions for winter wheat production in China, accounting for almost half of national wheat harvest areas^29^. Observations showed the trend of significant warming occurred in the North China Plain, especially during the growing season of winter wheat^30^, which affected winter wheat production in the area^3,4,31,32^. A number of studies have examined the impacts of warming and CO_2_ enrichment on winter wheat yield in the region^33–35^, however, it is still unclear how winter wheat would respond if both air temperature and CO_2_ concentration increase in the future.

The primary objectives of this study are: 1) to examine, using the open top chamber method with supplemental infrared heating, the growth and yield responses of winter wheat to a combination of elevated CO_2_ concentration and warming, 2) to investigate the mechanism of the combined impacts in the North China Plain, and 3) to reveal the changes of winter wheat hazard environmental factors under future climate change in order to provide scientific evidence for policy makers to make strategic plan for future winter wheat production in the North China.

## Results

### Developmental stage

The post-winter developmental stages of winter wheat for ECTI advanced significantly compared to those for CK (Table 1). For example, the date for jointing advanced by 16–19 days (normally it occurred late March) and maturity advanced by 15–18 days in three experimental seasons. During the winter period, it was observed that the leaves of the plants grown in the ECTI chambers remained green but those in the CK chambers became brown.

**Table 1:** The dates (Gregorian day) of main developmental stages of winter wheat under CK and ECTI.

Growing season	Treatment	Jointing stage	Heading stage	Flowering stage	Maturity
2013–2014	CK	86 ± 0	108 ± 0b	118 ± 0	159 ± 0
	ECTI	70 ± 0a*	92 ± 1b*	101 ± 1c*	141 ± 0*
2014–2015	CK	85 ± 0	114 ± 0a	120 ± 0	160 ± 0
	ECTI	67 ± 0b*	94 ± 1ab*	105 ± 1a*	145 ± 0*
2015–2016	CK	89 ± 0	111 ± 1ab	118 ± 0	158 ± 0
	ECTI	70 ± 1a*	94 ± 2a*	103 ± 1b*	141 ± 0*

Notes: Different lowercases indicate significant differences among different years within the same temperature treatment (P < 0.05); ^*^indicates significant difference between CK and ECTI within the same period (*P* < 0.05). The same as below.

### Winter wheat growth

**Figure 1:**
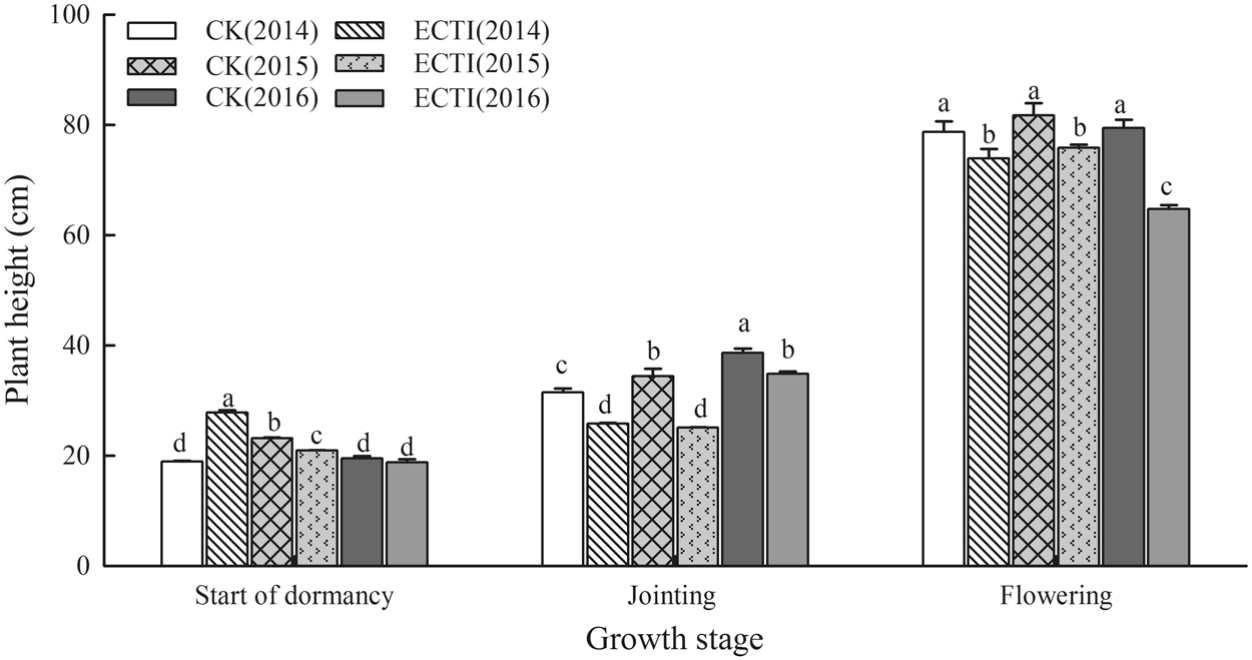
Canopy height of winter wheat grown under ambient (CK) and elevated (ECTI) conditions at different growth stages. Error bars indicate the standard deviation and letters over the bars at each stage are significantly different at the *P* < 0.05 level.

Plant height at various stages in each growing season is shown in Fig. 1. At the start of dormancy, plant height for ECTI was 9 cm higher than that for CK in the first growing season but slightly lower than that in CK in the next two seasons due to delayed sowing for ECTI. Although the plants in the ECTI chambers grew earlier, plant height was lower than that under CK at same developmental stages after jointing stage.

**Figure 2:**
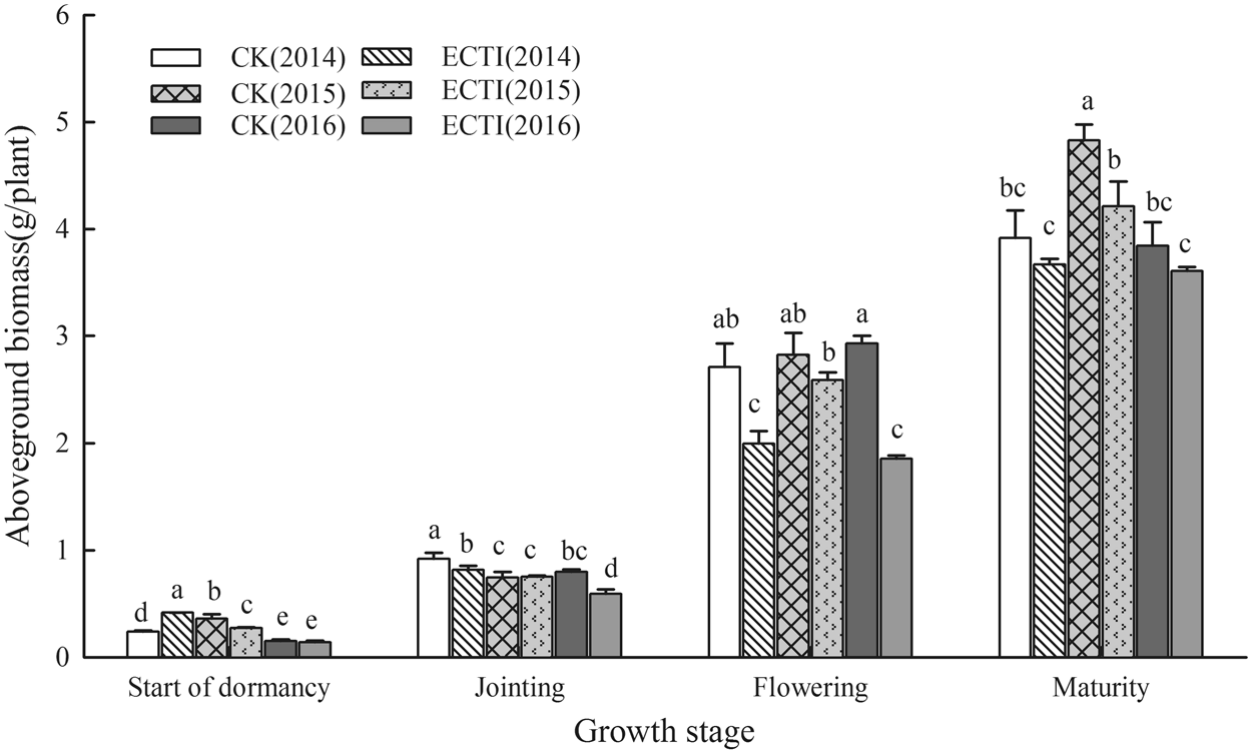
Above-ground dry matter of winter wheat grown under ambient (CK) and elevated (ECTI) conditions at different growth stages. Error bars indicate the standard deviation and letters over the bars at each stage are significantly different at the *P* < 0.05 level.

Aboveground dry matter accumulation for both treatments showed a similar trend as plant height (Fig. 2). Before dormancy, wheat plants grown in the ECTI chambers accumulated more dry matter than those in the CK chambers during the first growing season. However, when the sowing date was postponed 12 days in the next two seasons, there was no significant difference between the treatments. After jointing stage, aboveground dry matter for the CK treatment was consistently higher than that for the ECTI treatment at same stages, but the difference apparently narrowed at maturity in 2013–2014 and 2015–2016.

**Figure 3:**
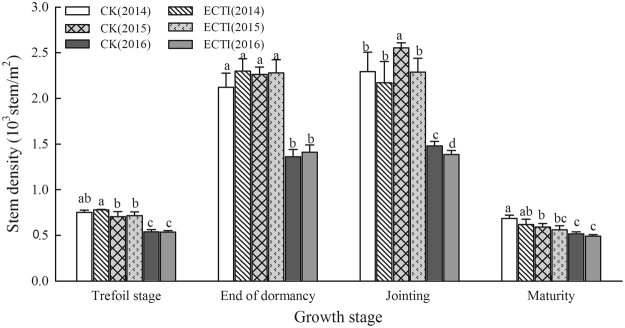
Stem density of winter wheat grown under ambient (CK) and elevated (ECTI) conditions at different growth stages. Error bars indicate the standard deviation and letters over the bars at each stage are significantly different at the *P* < 0.05 level.

Stem density data suggests that the combined effect of elevated CO_2_ concentration and warming on density varied with developmental stages (Fig. 3). Plants in ECTI had more tillers at the end of dormancy but in reverse at jointing stage. However, the differences in stem density between ECTI and CK at maturity became negligible for all three growing seasons.

### Yield components and grain yields

Yield components and grain yields of winter wheat for the ECTI and CK treatments in the three experiment seasons are shown in Table 2. The number of kernels and kernel weight were greatly affected by elevated CO_2_ concentration and warming. The number of kernels in an ear for ECTI decreased, on average, by 10–31% compared to that for CK in three seasons. By contrast, 1000-kernel weight for the ECTI treatment increased by 8–9%. Effective panicles per unit land area under ECTI were slightly more than those under CK without statistically significant difference between them. The positive effect of a combination of elevated CO_2_ concentration and warming on 1000-kernel weight partially offset the negative effect on the number of kernels in an ear, thus, the grain yields for ECTI were not reduced significantly compared to CK except in the 2014–2015 season.

**Table 2:** Grain yields and yield components of winter wheat grown under a combination of elevated CO_2_ concentration and warming (ECTI) and ambient conditions (CK), (Notes as Table 1).

Growing season	Treatment	Number of spikelet per ear	Number of effective panicles (panicle/m^2^)	Number of kernels per ear	The 1,000-kernel weight (g)	Yield (g/m^2^)
2013–2014	CK	16.0 ± 0.5a	582.0 ± 6.0a	40.5 ± 2.0b	49.5 ± 1.1ab	649.8 ± 35.3b
	ECTI	15.7 ± 0.6a	529.3 ± 30a*	35.7 ± 0.1b*	53.4 ± 0.5a*	630.6 ± 45.7a
2014–2015	CK	15.9 ± 0.4a	572.0 ± 33.9a	49.8 ± 1.2a	50.1 ± 1.2a	736.8 ± 13.3a
	ECTI	15.6 ± 0.3a	557.0 ± 24.3a	34.5 ± 2.1b*	54.5 ± 1.3a*	626.1 ± 55.2a*
2015–2016	CK	16.4 ± 0.4a	503 ± 18.7b	43.1 ± 2.5b	47.7 ± 0.6b	734 ± 25.7a
	ECTI	15.6 ± 0.3a*	480 ± 13.7b	38.9 ± 1.8a	51.6 ± 1.0b*	697.3 ± 17.3a

### The changes of environmental factors in wheat growth

The ECTI treatment with the daily mean temperature increased by 2.5–2.8 °C over the entire growing season only elevated the average temperature during the periods of emergence to dormancy in winter wheat, as shown in Table 3, and postponed sowing could eliminate this effect on period from emergence to start of dormancy. The significant advance of post-winter growing phase in ECTI to a cooler period of natural season caused lower daily temperature under which winter wheat grew, particularly over the period from the end of dormancy to start of jointing stage. Mean temperature over the entire post-winter growth period in ECTI was 0.8–1.5 °C lower than that in CK. Meanwhile, the average minimum temperature during critical periods of spikelet and floret development and at anthesis for ECTI were much lower than that for CK (Table 4), and the average maximum temperature from booting to anthesis had not increased. At the same time, the significant advance of post-winter growth stages to earlier dates for ECTI also led to less incident solar radiations during same growth period than that in CK. Accumulated photosynthetic active radiations (PAR) over the post-winter growing period for ECTI decreased 10–13% compared to that for CK in three experimental seasons (Table 5).

**Table 3:** Average temperature over the different developmental periods of winter wheat (°C) under two treatments (Notes as Table 1).

Growing season	2013–2014		2014–2015		2015–2016	
	CK	ECTI	CK	ECTI	CK	ECTI
Emergence–start of dormancy	6.4 ± 0.3b	7.7 ± 0.7a*	6.5 ± 0.2b	7.3 ± 0.6a	7.3 ± 0.3a	6.6 ± 0.3a*
Dormancy period	−2.3 ± 0.1a	−0.6 ± 0.5a*	−2.4 ± 0.2a	−0.6 ± 0.4a*	−3.8 ± 0.2b	−2.3 ± 0.2b*
End of dormancy–Jointing	8.0 ± 0.4a	5.0 ± 0.5a*	6.3 ± 0.3c	4.0 ± 0.5ab*	7.1 ± 0.2b	4.2 ± 0.4b*
Jointing–Heading	15.2 ± 0.4a	14.6 ± 0.3a	13.0 ± 0.3b	11.8 ± 0.2c*	15.0 ± 0.2a	13.3 ± 0.6b*
Heading–mature	20.8 ± 0.4a	20.2 ± 0.5a	21.0 ± 0.2a	19.5 ± 0.3a*	20.7 ± 0.3a	20.3 ± 0.6a
End of dormancy–mature	16.3 ± 0.4a	15.5 ± 0.5a	15.0 ± 0.2b	13.6 ± 0.3b*	15.0 ± 0.3b	13.5 ± 0.5b*

**Table 4:** The average minimum temperature (Tmin1, Tmin2) in consecutive 7 days with the centre on jointing date and flowering date, and the average maximum temperature (Tmax) from booting date to flowering date under CK and ECTI (Notes as Table 1).

Growing season	Treatment	Tmin1	Tmin2	Tmax
2013–2014	CK	8.3 ± 0.4a	10.7 ± 0.5c	25.2 ± 0.6a
	ECTI	0.9 ± 0.6a*	8.6 ± 0.5ab*	25.0 ± 0.7a
2014–2015	CK	5.5 ± 0.3b	11.6 ± 0.5b	26.2 ± 1.0a
	ECTI	−1.6 ± 0.7b*	7.8 ± 0.9b*	24.6 ± 0.4a
2015–2016	CK	6.2 ± 0.5b	12.9 ± 0.4a	25.6 ± 0.5a
	ECTI	−2.6 ± 0.9b*	9.6 ± 0.6a*	25.1 ± 0.6a

**Table 5:** Accumulated photosynthetic active radiation (PAR, MJm^−2^) and length from the end of dormancy to mature (days) for each treatment.

Treatment	2014		2015		2016	
	PAR	Days	PAR	Days	PAR	Days
CK	835.1 ± 0.0	98 ± 0.0	930.2 ± 0.0	102 ± 0.0	896.5 ± 0.0	103 ± 0.0
ECTI	707.4 ± 0.0	93 ± 0.0	860.6 ± 0.0	105 ± 0.0	795.5 ± 0.0	105 ± 0.0

## Discussion

The three-year experiment showed that the increase of 2.5–2.8 °C in daily average temperature coupled with 600 μmol mol^−1^ CO_2_ concentration over an entire growing season significantly shortened the length of growth stage of winter wheat, that was completely attributed to shortening dormancy due to temperature rise over the dormancy period (Table 3). The variance analysis among the effects of temperature treatments and different years on different environmental factors of main developmental stages were shown in Supplementary Table S1. Advanced days for jointing stage and maturity under ECTI were, on average over three years, 17.7 and 16.6 days, respectively, i.e. shifting a similar length, which indicated the post-winter growth duration did not shorten but the dates of developmental stages shifted. This result is consistent with the findings from previous warming experiments^27,36^, historical observations on the phenological change of winter wheat in the studied region^31,37^ and general observations^38^.

The post-winter growth stages of winter wheat largely advanced and shifted to a cooler period of nature season under a daily mean temperature increase of 2.5–2.8 °C over the entire growing season. As a result, the mean temperatures over post-winter growing periods in ECTI not only didn’t rise, but decreased. Similar results from warming experiments have been reported previously^25^. The relatively lower temperature from the end of dormancy to anthesis in this experiment directly decreased the growth rate and weakened the stimulating effect of high CO_2_ concentration on photosynthesis, as reported from previous studies^15–17^. Meanwhile less incident solar radiations during same growth period would limit photosynthetic capability and partly offset the stimulating effect of high CO_2_ concentration on photosynthesis. Lower temperature and less solar radiation during growth stage led to shorter plant height and less aboveground biomass (Figs 1–2) despite the elevated CO_2_ concentration of 600 μmol mol^−1^. Nevertheless, Fig. 2 also showed the effect of elevated CO_2_ concentration on wheat growth became more obvious in ECTI after anthesis when air temperature was relatively higher, just as reported by Amthor^7^. The result of our experiment also suggested that appropriate late sowing could mitigate the effect on growth of elevated temperature before winter and avoid overgrowth of winter wheat.

The number of kernels per ear under ECTI dramatically reduced (Table 2) in this study, that was mainly due to abortion of grains at the top of ear to fill. Such an effect of combined impacts of increased temperature and CO_2_ on kernel numbers per ear of winter wheat has been reported by Mitchell^24^, where the reduction of kernel numbers per ear was interpreted as exposing to high temperature during critical periods between booting and anthesis. However, in our study, we had observed that the average maximum temperature from booting to anthesis in ECTI was lower than that in CK (Table 4). By contrast, winter wheat grown in ECTI experienced extreme low temperatures due to significant advance of developmental stages during the periods of floret development, which generally last from the beginning of jointing to anthesis in North China Plain^39^. It has been reported that temperature close to 0 °C during the period of floret initiation inhibited floret development and caused degradation of florets that have already formed, and temperature lower than 9.5 °C at anthesis could result in floret infertility^19,40^. As shown in Table 4, the average minimum temperatures in consecutive 7 days at jointing in ECTI were below 0 °C in 2015 and 2016, and the average minimum temperatures in consecutive 7 days at anthesis in ECTI were lower than 9.5 °C in 2014 and 2015. Therefore the extreme low temperature during the critical period might be the main reason for dramatic reduction of kernel numbers per ear. Ottman^22^ has reported on similar observations and indicated that cold temperatures during critical stages of floret development were lethal to grain development and led to significant decrease of kernels per spike.

It appears that increase of mean daily temperature for growing season under a certain range didn’t seriously threaten the winter wheat production in North China Plain^34^, because of adaptive advance of post-winter growth stages. But the changes of those environmental factors would have negatively effects on the growth and yield of winter wheat. Winter wheat would suffer from low temperature during critical period of floret development, which might lead to large reduction of kernel numbers per ear. This finding was inconsistent with the results of Cai^25^ and Tian^27^, which reported that warming affected winter wheat yield in East China, where winter is shorter and warmer than in North China, and climate warming may reduce the frost/chilling stress to wheat growth. This indicates that the effects of climate warming on winter wheat in North China might be different from that in East China, as well as in South China. We should pay more attention to low temperature damage on winter wheat yield in the context of future climate change in study region instead of high temperature stress, and new cultivars that can resist low temperatures during critical period should be bred to adapt to the change.

In conclusion, the three-year experiment showed that the future climate change would affect seriously the winter wheat growth and grain yield. Moreover, the winter wheat would suffer from low temperature during critical period of floret development and the grain number per ear was reduced by 10–31% in three years under combined impact of elevated CO_2_ concentration with a daily mean temperature increase of 2.5–2.8 °C over the entire growing season. Low temperature hazards coming with climate warming might become an important threat to the stable production of winter wheat in North China Plain.

## Methods

### Site description

This study was conducted at the Gucheng Ecological and Agro-meteorological Experimental Station of the Chinese Academy of Meteorological Sciences (Dingxing County, Hebei Province, 39^o^08’N, 115^o^40’E) from October 2013 to June 2016, covering three growing seasons for winter wheat. It is in the North China Plain, with annual mean temperature of 11.9 °C and mean precipitation of 552 mm (2000–2014). Approximately, 70% of annual precipitation occurs between June and September. The season for growing winter wheat normally is from mid-October to mid-June the following year in this area. The mean monthly temperatures over experimental seasons are given in Table 6. There is a long dormant period from the end of November to the beginning of March, after which winter wheat begins to grow rapidly. The soil is classified as a typical cinnamon soil (Torrifluvents based on the USDA soil classification), with organic matter content of 11.0 g kg^−1^, total nitrogen content of 1.03 g kg^−1^ and pH of 8.1 in the topsoil layer (0–30 cm depth).

**Table 6:** Mean monthly temperatures during experimental seasons (°C).

	Nov.	Dec.	Jan.	Feb.	Mar.	Apr.	May
Average year	4.3	−1.9	−3.9	−1.2	6.0	14.3	20.6
2013–2014	4.2	−2.1	−2.9	−1.3	8.3	15.7	20.9
2014–2015	4.2	−2.8	−2.8	−0.2	7.7	14.6	20.1
2015–2016	3.2	−1.4	−5.7	−0.3	7.0	16.0	19.9

### Experimental design

The experiment was conducted using a group of open top chambers. There are two treatments with three replicates each: elevated both temperature and CO_2_ concentration (hereafter ECTI) and a control (i.e. ambient CO_2_ and temperature, hereafter CK). The increases in temperature and atmospheric CO_2_ concentration are based on the change projections for the 2070 s in the Plain^30,41^. Daytime (08:00–20:00) temperature increase was set at 1–2 °C and night-time (20:00–08:00) temperature increase was set at 3.5–4.5 °C in the ECTI treatment. Elevated CO_2_ concentration was set at 600 μmol mol^−1^.

**Figure 4:**
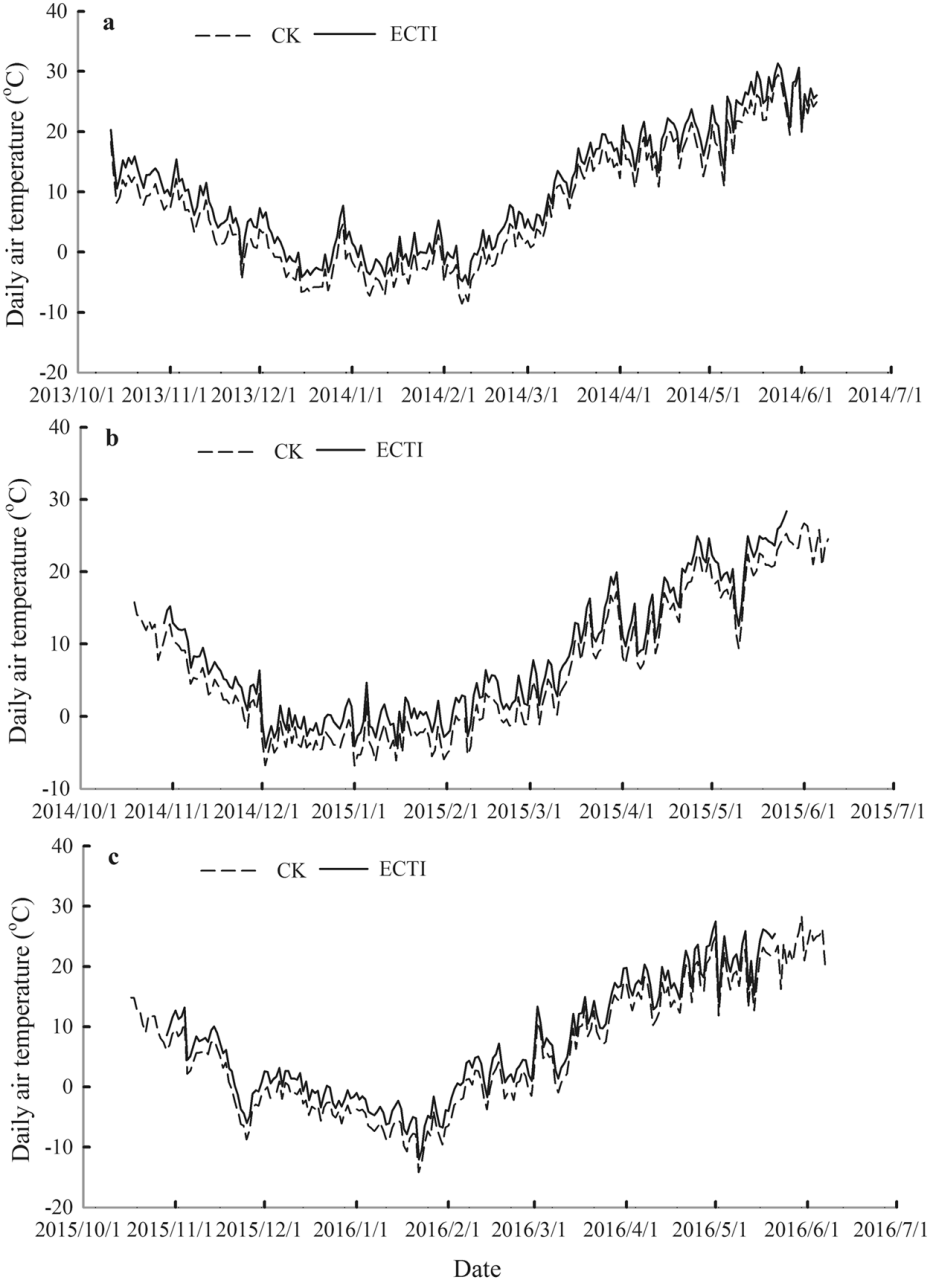
Dynamics of daily mean temperature in the control and elevated chambers during the growing period of winter wheat. (**a**) 2013–2014; (**b**) 2014–2015; (**c**) 2015–2016.

The octagonal chambers were made of plastic-steel glass. Each chamber is 2.5 m in height with an area of 10 m^2^ and ventilated by a 2000 m^3^ h^−1^ centrifugal fan at uniform speed through a PVC pipe system. To eliminate the passive warming effect during daytime by the chamber itself, the glass panels on the north and northeast face of the chambers used for CK were removed and an opening of 25 cm in height at the base of both the east and west sides of the chambers was left. The passive warming effect in chambers for ECTI was eliminated partially by means of ventilation system and the temperature difference with the CK chambers during daytime was compliant with the experimental design. During night-time temperature increased through four infrared heaters (850 W) installed on the frame at the top of a chamber for the ECTI treatment. The infrared heaters were positioned so as not to shade the crops. A temperature sensor with a naturally ventilated radiation shield was installed at the centre of each chamber and in field outside chambers at 50 cm above the ground. Temperature was recorded at a 10-min interval. Dynamics of daily average temperature in the chambers during the growing seasons are shown in (Fig. 4). The average increments of daytime, night-time and daily temperatures over growing seasons for ECTI are shown in Table 7. The temperature differences in chambers for CK with ambient outside chamber during daytime were of 0.4 ± 0.4 °C, 0.5 ± 0.6 °C and 0.5 ± 0.5 °C in three experimental seasons respectively.

**Table 7:** Average temperatures and concentrations of CO_2_ in the chambers during the periods of heating and CO_2_ fumigation (Mean ± SD), notes as Table 1.

Growing season	Treatment	Night-time	Daytime	Daily mean	[CO_2_]
2013–2014	CK	1.3 ± 0.3a	10.2 ± 0.3a	5.8 ± 0.3a	397 ± 15
	ECTI	5.4 ± 0.5a*	11.7 ± 0.4a*	8.6 ± 0.4a*	612 ± 49
2014–2015	CK	0.6 ± 0.3b	9.9 ± 0.6a	5.3 ± 0.4ab	399 ± 13
	ECTI	4.5 ± 0.6a*	11.5 ± 0.6a*	7.9 ± 0.5ab*	603 ± 55
2015–2016	CK	1.0 ± 0.3ab	8.5 ± 0.4b	4.8 ± 0.3b	401 ± 17
	ECTI	4.8 ± 0.4a*	10.1 ± 0.5b*	7.3 ± 0.4b*	617 ± 59

Carbon dioxide fumigation was only activated during the daytime and no CO_2_ fumigation when winter wheat was dormant in winter. Real-time CO_2_ concentration in chambers was monitored by an infrared gas analyser (QGS-08C, BAIF-Maihak, Beijing, China). CO_2_ emission was adjusted manually in real time to maintain the CO_2_ concentration at the 600 μmol mol^−1^ level.

Winter wheat (cv. Tanmai-98) seeds were sown on 10 October 2013 for both treatments. To design a growing environment for winter wheat that accumulated temperatures before dormancy could have a same value for both treatments, which would represent one of the adaption measures to future climate change, we sew them on 22 October for treatment ECTI while on 10 October for CK in the 2014–2015 and 2015–2016 seasons. The seed rate was 230 kg ha^−1^ in the first two growing seasons and 180 kg ha^−1^ for the third season.

Precipitation during the growth period for the three experimental seasons was 71, 105 and 124 mm, respectively. There were five irrigation events as common practice in the region: after sowing, before wheat dormancy in winter, before jointing, at booting and at the start of grain filling. Irrigation intensity for each event was 100 and 120 mm for CK and ECTI, respectively, that could ensure adequate water supply for winter wheat in CK and ECTI. 600 kg ha^−1^ of di-ammonium phosphate (DAP, (NH4)_2_HPO_4_) was applied as basal fertiliser and then 250 kg ha^−1^ of urea and DAP each by topdressing after the end of dormancy.

### Measurements

Canopy height, density and aboveground biomass were measured at trefoil stage (three leaves unfolded), start and end of dormancy, jointing stage, flowering stage and maturity. Ten plants were randomly chosen to measure their heights from the surface of soil to the top. The number of plants in a row with 1 m length were counted and then converted to plants m^−2^. For each sampling ten plants randomly chosen from each chamber were dug out, after cutting off the roots, the aboveground parts were dried up for 48-hours in an oven with a constant temperature of 80 °C and then weighed for dry matter. At maturity, plants in a 2 m^2^ quadrate from each chamber were harvested for yield components (kernel weight, total ear number, number of effective panicles and number of non-productive ears), grain yield, and aboveground dry matter. 40 main stems randomly picked up from the harvested plants were used to count the number of kernels and number of spikelet in each ear. Photosynthetically active radiation (PAR) was observed in one chamber by a sensor (kipp & zonen) and recorded by data logger at 1 minute interval.

### Statistical analysis

A one-way ANOVA method was applied to test the significant differences of the average grain yield, yield components, canopy height, stem density and dry matter accumulation between the treatments. The significant differences between the treatments were compared with the least significant differences (L.S.D) at 5 and 1% levels of probability. All statistical analysis was performed with SPSS (SPSS, Inc., 1999, Chicago, USA, www.spss.com).

### Data availability

All data generated or analysed during this study are included in this published article.

## Electronic supplementary material

Supplementary Table S1
