# Supplementary material for: Combined effects of elevated temperature and CO2 enhance threat from low temperature hazard to winter wheat growth in North China
Source: Sci Rep. 2018 Mar 12;8:4336. doi: 10.1038/s41598-018-22559-4 (PMC5847586; doi:10.1038/s41598-018-22559-4)
Supplement: Supplementary file 1 — Supplementary Table S1 [file 41598_2018_22559_MOESM1_ESM.pdf]

Combined effects of elevated temperature and CO_2 enhance threat from low temperature hazard to winter wheat growth in North China

Kaiyan Tan[1], Guangsheng Zhou[1,2, *], Xiaomin Lv[1], Jianping Guo[1], Sanxue Ren[1]

[1] State Key Laboratory of Severe Weather, Chinese Academy of Meteorological Sciences, Beijing, 100081, China

[2] Collaborative Innovation Center on Forecast Meteorological Disaster Warning and Assessment, Nanjing University of Information Science & Technology, Nanjing, 210044, China

[*]Corresponding: zhougs@cma.gov.cn

Table S1. One-way ANOVA (*F* value) analyses among the effects of temperature treatments and different years on different environmental factors, yield components and dates (gregorian day) of main developmental stages.

Variances		Treatment		Years	
		df	*F* value	*df*	*F* value
Average temperatures	Night-time	1	267.03**	2	0.23
	Daytime	1	15.46**	2	4.91**
	Daily mean	1	92.37**	2	0.84
Average temperature of different developmental periods	Emergence-start of dormancy	1	2.45	2	0.08
	Dormancy period	1	17.92**	2	5.53*
	End of dormancy-Jointing	1	66.32**	2	1.23
	Jointing-Heading	1	4.65*	2	16.27**
	Heading- mature	1	16.23**	2	0.2
	End of dormancy-mature	1	9.04**	2	8.74**
Dates of main developmental stages	Jointing stage	1	473.25**	2	0.22
	Heading stage	1	321.00**	2	0.25
	Flowering stage	1	406.22**	2	0.23
	Maturity	1	526.32**	2	0.19
Yield components	Number of spikelet per ear	1	5.85**	2	0.46
	Number of effective panicles	1	2.47	2	12.23**
	Number of kernels per ear	1	22.50**	2	0.86
	The 1,000-kernel weight	1	35.20**	2	1．93
	Yield	1	5.67**	2	3.56
PAR		1	14.40**	2	7.48**
days of dormancy to mature		1	0.00	2	25.92**
Tmin1		1	117.00**	2	0.81
Tmin2		1	40.33**	2	1.62
Tmax		1	6.45**	2	0.31

Notes: *, **represent statistically significant at $P<0.05$ and 0.01; Tmin1 and Tmin2 respectively referred to the average minimum temperature in consecutive 7 days with the centre on jointing date and flowering date; Tmax, the average maximum temperature from booting date to flowering date.
